# Supplementary material for: Identification of photoperiod-induced specific miRNAs in the adrenal glands of Sunite sheep (Ovis aries)
Source: Front Vet Sci. 2022 Jul 22;9:888207. doi: 10.3389/fvets.2022.888207 (PMC9354845; doi:10.3389/fvets.2022.888207)
Supplement: Supplementary file 1 [file Data_Sheet_1.ZIP › Supplementary file/miRDeep2 file.zip!/miRDeep2 file/Know_Structure/SPLP42A1/oar-mir-374a.pdf]

miRBase precursor	: oar-mir-374a
Total read count	: 24100
oar-miR-374a read count	: 20293
remaining reads	: 3807

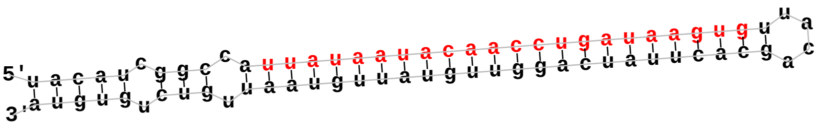

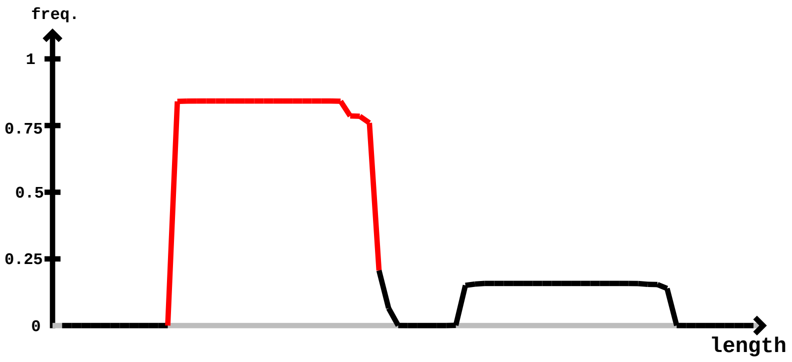

oar-miR-374a

```
5'- uacaucggccauuauaauacaaccugauaaguguuacagcacuuaucagguuguauuguaauugucugugua  -3'    exp
    (((((.(((.((((((((((((((((((((((((....)))))))))))))))))))))))).))).)))))   reads      mm        sample
    ...........uuGuaauacaaccugau............................................   1          1         seq
    ...........uuauaauacaaccugau............................................   17         0         seq
    ...........uuauaauacaaccugaA............................................   1          1         seq
    ...........uuauUauacaaccugaua...........................................   7          1         seq
    ...........uuaCaauacaaccugaua...........................................   12         1         seq
    ...........uuaAaauacaaccugaua...........................................   8          1         seq
    ...........uuauaauacaaccugGua...........................................   7          1         seq
    ...........Cuauaauacaaccugaua...........................................   12         1         seq
    ...........uuauaauacaaccugauU...........................................   12         1         seq
    ...........uuauaauacaaccugaua...........................................   1268       0         seq
    ...........uCauaauacaaccugaua...........................................   1          1         seq
    ...........uuauaauacaUccugaua...........................................   2          1         seq
    ...........uuauaauacaaccugauaa..........................................   27         0         seq
    ...........uuauaauacaaccugauaag.........................................   591        0         seq
    ...........uuaCaauacaaccugauaag.........................................   4          1         seq
    ...........uuauaauacaaccugGuaag.........................................   3          1         seq
    ...........uuauaauacaaccugauaagu........................................   13187      0         seq
    ...........uGauaauacaaccugauaagu........................................   1          1         seq
    ...........uuaCaauacaaccugauaagu........................................   136        1         seq
    ...........uuauaauacaaccugauaagug.......................................   3371       0         seq
    ...........uGauaauacaaccugauaagug.......................................   1          1         seq
    ...........uuaCaauacaaccugauaagug.......................................   35         1         seq
    ...........uuauaauacaaccugauaagugu......................................   1552       0         seq
    ...........uuauaauacaaccugauaaguguu.....................................   2          0         seq
    ............uauaauacaaccugaua...........................................   3          0         seq
    ............uauaauacaaccugauaag.........................................   1          0         seq
    ............uauaauacaaccugauaagu........................................   8          0         seq
    ............uauaauacaaccugauaagug.......................................   3          0         seq
    ............uauaauacaaccugauaagugu......................................   10         0         seq
    ............uauaauacaaccugauaaguguu.....................................   2          0         seq
    .............auaauacaaccugauaagu........................................   4          0         seq
    .............auaauacaaccugauaagug.......................................   1          0         seq
    ..............uaauacaaccugauaagu........................................   1          0         seq
    ..............uaauacaaccugauaagugC......................................   1          1         seq
    ...............aauacaaccugauaagugu......................................   1          0         seq
```

oar-miR-374a

uacaucggccauuauaauacaaccugauaaguguuacagcacuuaucagguuguauuguaauugucugugua			
....acuuaucagguuguauugu.............	2	0	seq
....acuuaucagguuguauugua............	1	0	seq
....acuuaucagguuguauuguaau..........	10	0	seq
....acuuaucagguuguauuguaauu.........	9	0	seq
....cuuaucagguuguauug..............	2	0	seq
....cuuaucagguuguauugu.............	8	0	seq
....cuuCucagguuguauugua............	3	1	seq
....cuuaucagguuguauugua............	64	0	seq
....cuuaucagguuguauuguaa...........	19	0	seq
....cuuaucagguuguauuguaau..........	330	0	seq
....cuuaucagguuguauuAuaau..........	1	1	seq
....cuuaucagguuAuauuguaauu.........	1	1	seq
....cuuaucagguuguauuguaauu.........	3181	0	seq
....cuuaucagguuguauuguaauug........	4	0	seq
....uuaucagguuguauugua............	1	0	seq
....uuaucagguuguauuguaau..........	7	0	seq
....uuaucagguuguauuguaauu.........	105	0	seq
....uuaucagguuguauuguaauugu.......	1	0	seq
....uaucagguuguauuguaau..........	4	0	seq
....uaucagguuguauuguaauu.........	53	0	seq
....uaucagguuAuauuguaauu.........	1	1	seq
